# The rabbit as an orthologous small animal model for APOBEC3A oncogenesis

**DOI:** 10.18632/oncotarget.25593

**Published:** 2018-06-12

**Authors:** Hélène C. Laude, Vincent Caval, Mohamed S. Bouzidi, Xiongxiong Li, Florence Jamet, Michel Henry, Rodolphe Suspène, Simon Wain-Hobson, Jean-Pierre Vartanian

**Affiliations:** ^1^ Molecular Retrovirology Unit, Institut Pasteur, CNRS UMR 3569, France; ^2^ Lanzhou Institute of Biological Products Co., Ltd (LIBP), subsidiary company of China National Biotec Group Company Limited (CNBG), Lanzhou 730046, China

**Keywords:** rabbit, animal model, APOBEC3A, cytidine deaminase, cancer

## Abstract

APOBEC3 are cytidine deaminases that convert cytidine to uridine residues. APOBEC3A and APOBEC3B enzymes able to target genomic DNA are involved in oncogenesis of a sizeable proportion of human cancers. While the *APOBEC3* locus is conserved in mammals, it encodes from 1–7 genes. *APOBEC3A* is conserved in most mammals, although absent in pigs, cats and throughout *Rodentia* whereas *APOBEC3B* is restricted to the *Primate* order. Here we show that the rabbit *APOBEC3* locus encodes two genes of which *APOBEC3A* enzyme is strictly orthologous to human APOBEC3A. The rabbit enzyme is expressed in the nucleus and the cytoplasm, it can deaminate cytidine, 5-methcytidine residues, nuclear DNA and induce double-strand DNA breaks. The rabbit APOBEC3A enzyme is negatively regulated by the rabbit TRIB3 pseudokinase protein which is guardian of genome integrity, just like its human counterpart. This indicates that the APOBEC3A/TRIB3 pair is conserved over approximately 100 million years. The rabbit *APOBEC3A* gene is widely expressed in rabbit tissues, unlike human *APOBEC3A*. These data demonstrate that rabbit could be used as a small animal model for studying APOBEC3 driven oncogenesis.

## INTRODUCTION

Cancer genomics has exploded our understanding of oncogenesis [1, 2]. Human cancer genomes harbor thousands to millions of mutations, as well as large numbers of insertions or deletions (indels) and chromosomal rearrangements [3, 4]. Human cancer genomes show signatures that provide clues as to etiological agents, *e.g*., benzo(a)pyrenes in tobacco smoke, ultraviolet light or endogenous APOBEC3A (A3A) or APOBEC3B (A3B) cytidine deaminases [3]. Six functional APOBEC3 cytidine deaminases (A3A-A3C and A3F-A3H) are encoded by the human genome and are made up of three related, but phylogenetically distinct zinc-finger domains referred to as Z1, Z2 and Z3 [5]. While the enzymes contain one or a pair of zinc finger domains, only the C-terminal domain is catalytically active. These enzymes convert cytidine residues to uridine in single-stranded DNA (ssDNA), a process referred to as DNA editing. Such substrate specificity distinguishes them from other cytidine deaminases involved in the metabolism of nucleotide precursors [6].

APOBEC3 proteins were first identified functionally as innate restriction factors of viruses such as human immunodeficiency virus, hepatitis B virus, herpesviruses and papillomavirus to mention a few [7–16]. It latter transpired that some A3 enzymes inhibit retrotransposition of SINEs and LINEs, endogenous retroviruses and can catabolize mitochondrial DNA leaked to the cytoplasm [17–20]. Viral restriction is linked to the fact that viral genomes are riddled with uridine residues that can result in so many mutations that genetic information is lost. The alternative is that the edited DNA is catabolized.

Human A3A and A3B are singular in that they can edit chromosomal DNA [20–24]. Interestingly, it is these two enzymes that can efficiently deaminate 5-methylcytidine residues (5MeC), which have long been described as cancer mutation hotspots [22, 25–27]. As a result of A3 editing of cytidine residues, uridine in DNA triggers damage repair response whose first step is the excision of the uracil base by uracil N-glycosylase (UNG). If both DNA strands are edited close to one another, double-strand DNA breaks (DSBs) are generated, a feature of all mammalian A3As [20, 22, 28–30]. By contrast, if the uracil base is copied before excision, it leads to a C->T substitution occurring frequently in the 5′TpC3′ or 5′CpC3′ context. Hence, this is the APOBEC3 editing signature that shows up in cancer genomes. Experimentally, A3A is more active than A3B and can induce DSBs [20–22, 29, 30]. In addition, the (in front of A3B) deletion allele is almost completely fixed in parts of Oceania [31] suggesting that A3B is dispensable for life and cancer.

The human Tribbles 3 pseudokinase (TRIB3) is part of the Rb-BRCA1-ATM network regulating genome integrity. Interestingly, TRIB3 is the only known negative interactor of human A3A [32]. TRIB3 is a nuclear protein that degrades A3A in a proteasome independent manner. Given that the A3A and A3B DNA mutator enzymes are etiological agents of human cancer, it would be useful to have a small animal model to study oncogenesis *in vivo*.

The *A3A* gene is found among most placental mammals [28], although not without a few exceptions. Sheep and dog have functional A3A enzymes but closely related pigs and cats are devoid of any homolog [28, 33]. *A3A* is completely lacking in *Rodentia* (mice, rats, guinea pigs and squirrels), which is unfortunate given the important of mice and rats as experimental models. As the rabbit is a commonly used small animal model, this prompted us to look at the rabbit *A3* locus. We show here that the rabbit genome encodes a strictly orthologous A3A enzyme which is the closest relative to primate counterparts. Furthermore, the enzyme is negatively regulated by the rTRIB3, just like its human counterpart that interferes with human A3A, highlighting a connection between the two proteins that has been conserved for more than 100 million years.

## RESULTS

### Rabbit genome encodes an *A3A* gene

**Figure 1:**
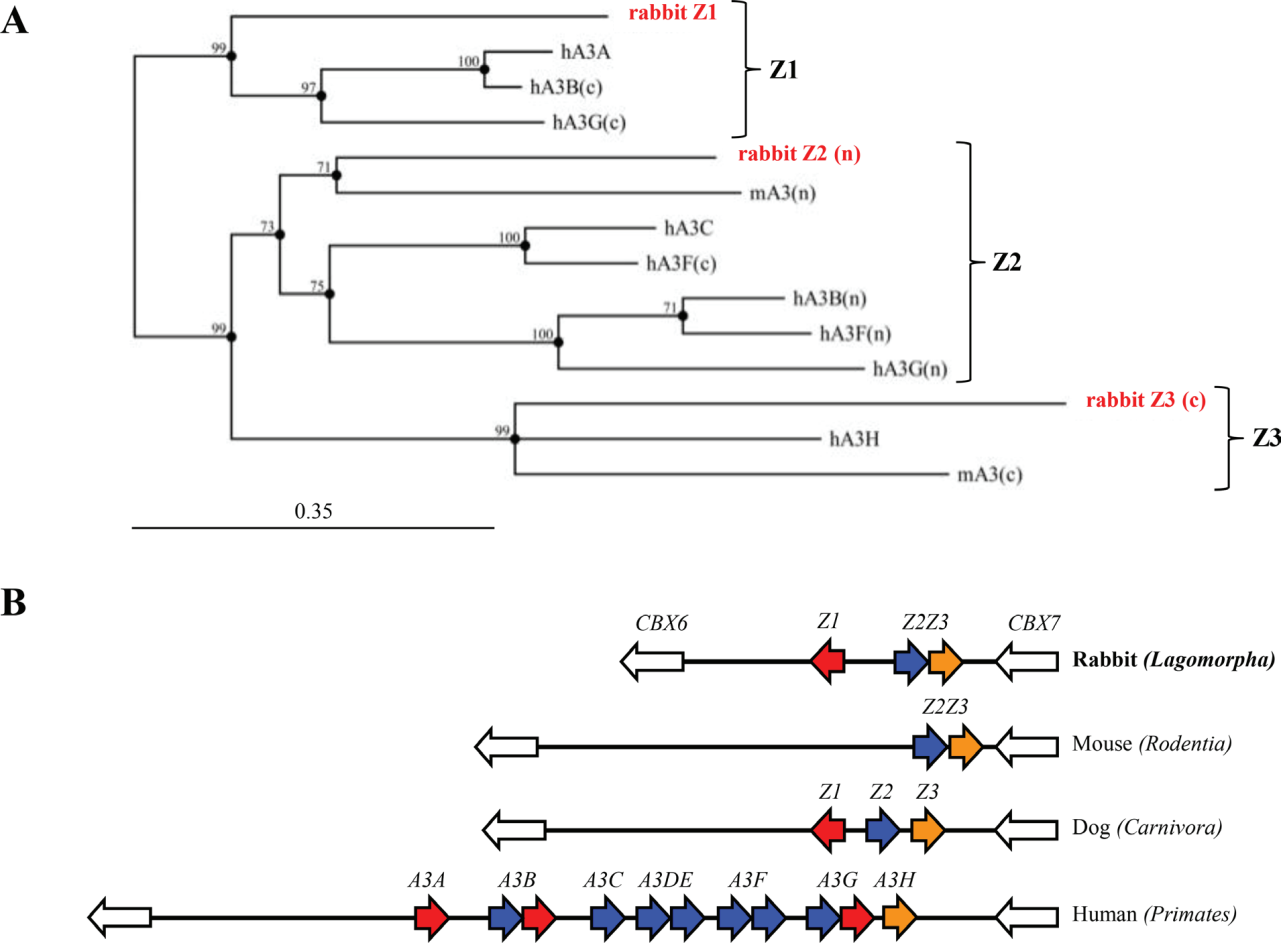
Rabbit *APOBEC3* locus (**A**) Phylogenic tree of three rabbit A3 protein domains along with mice (mA3) and human (hA3A, hA3B, hA3C, hA3F and hA3G) A3 protein sequences constructed using the Neighbor-joining method with the MCC Main Workbench 7.0.2 software. Numbers correspond to bootstrap values inferred from 1,000 replicates. Bootstrap values under the threshold of 70% are not shown. Letters n and c in brackets refer to the N-terminal or C-terminal zinc-finger domains. (**B**) To scale representation of the rabbit, mouse, dog and human *A3* loci bounded by the *CBX6* and *CBX7* represented by white arrows. The Order is given in brackets.

Through extensive BLAT, BLAST and EST screening, we identified three complete APOBEC3 like Zn-finger domains on rabbit chromosome 4. Potential exons/introns junctions were verified by PCR and sequencing of cDNA fragments (data not shown). As shown in Figure 1A, each rabbit sequence clusters with a Z1, Z2 or Z3 domain for human and mice APOBEC3 enzymes. All *A3* loci to date are bounded by *CBX6* and *CBX7* and the rabbit locus was no exception indicating that the entire locus was probably correctly assembled (Figure 1B). The two rabbit A3 genes are organized in a head-to-head manner reminiscent of the *Carnivora* order. The rabbit Z2 and Z3 domains are closely juxtaposed and proximal to *CBX7*. As the unique mouse A3 enzyme is made of both Z2 and Z3 domains and is closely related to the rabbit Z2 and Z3 domains, we suspected that the two Z2Z3 domains could belong to the same enzyme in rabbit. We confirmed by PCR amplification of rabbit cDNA across the Z2Z3 junction that the same was true (data not shown). Accordingly, the rabbit genome encodes two APOBEC3 genes that will be referred to as A3Z1 (rA3A) and A3Z2Z3 (rA3Z2Z3). As shown in Figure 1A, the rabbit A3Z1 (rA3A) is homologous to the human A3A and A3B cancer related genes.

### Rabbit A3A enzyme is closely related to human A3A

**Figure 2:**
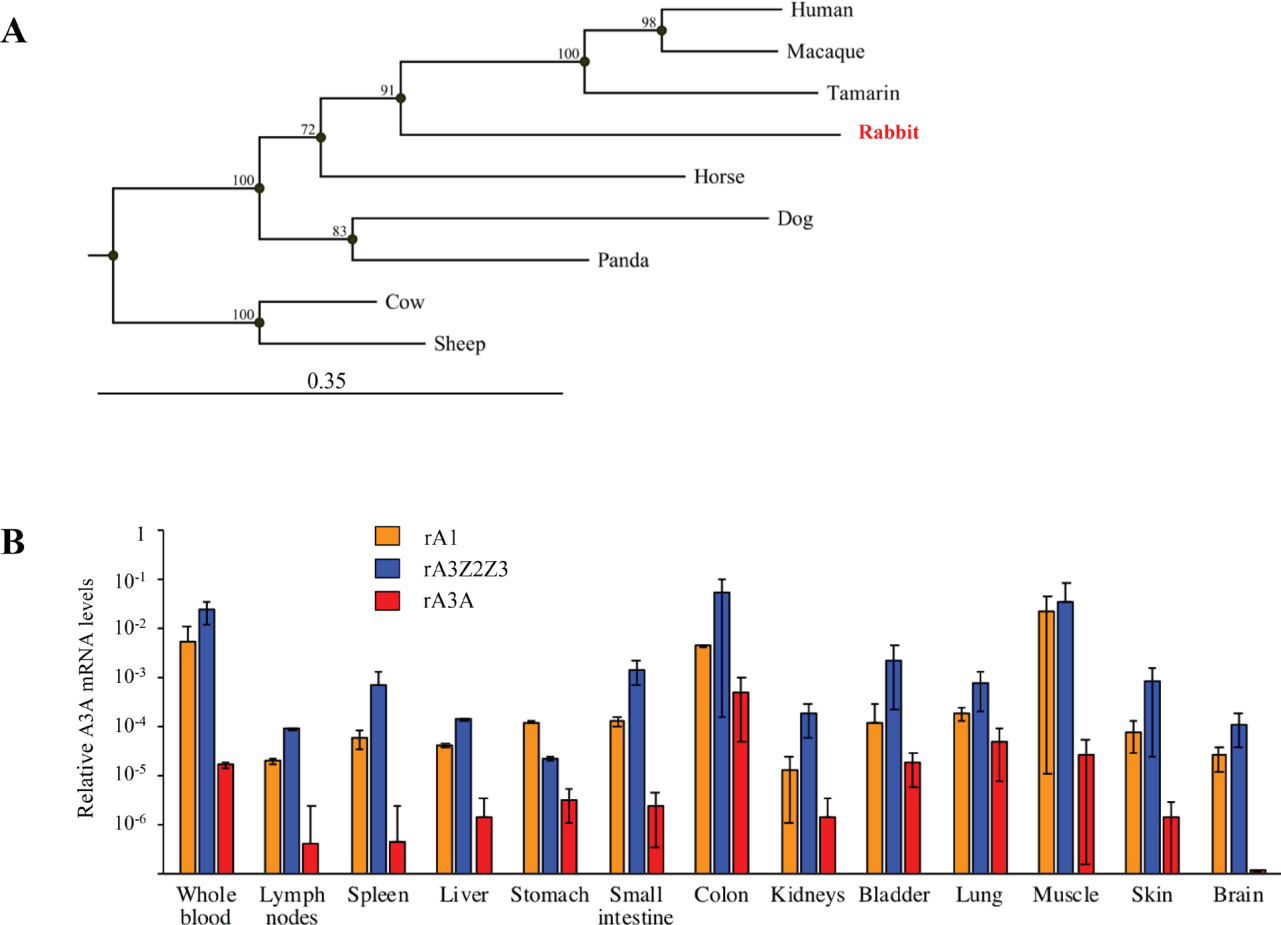
Rabbit A3A protein and gene expression (**A**) Phylogeny of a selection of mammalian A3A protein sequences constructed using the Neighbor-joining method with the CLC Main Workbench 7.0.2 software (Qiagen). Numbers correspond to bootstrap values inferred from 1,000 replicates. (**B**) RT-PCR quantification of *rA1* (orange), *rA3Z2Z3* (blue) and *rA3A* (red) mRNA in blood and rabbit tissues. Results were normalized to rabbit *EF1α* reporter gene expression.

The rabbit A3A amino acid sequence was aligned with those of 8 mammalian enzymes revealing subtle differences distinguished rabbit from the human APOBEC3 sequence (Supplementary Figure 1). The rabbit sequence is colinear with all others and starts with a strong Kozak AUG sequence, unlike the human gene where translation is initiated at two sites [30, 34]. Indels generally map to loops although there is a deletion of some β2 sheet residues. The rabbit sequence encodes all the key residues found in the catalytic site of A3A enzymes as well as I128 that is characteristic of all Z1 domains [5, 33]. Phylogenetic analysis of these protein sequences showed that the rA3A sequence was closest to those of the *Primate* A3A group being supported by a good bootstrap value (Figure 2A). Phylogenetic analyses of the rabbit Z2Z3 (rA3Z2Z3) protein sequences revealed that the Z3 sequence was an outlier to the primate group of sequences (Supplementary Figure 2A), much like the rabbit Z1 sequence (Figure 1A). By contrast, there was not enough sequence resolution to position the rabbit Z2 domain (Supplementary Figure 2B).

To study gene expression, we performed quantitative PCR on the two APOBEC3 cDNAs from a large number of rabbit tissues along with rabbit APOBEC1 (rA1) as control. Although rA1 deaminates cytidine residues primarily in dsRNA, it has been implicated in oncogenesis [35] while its murine counterpart edits both dsRNA and ssDNA [36]. Data were normalized to the housekeeping *EF1α* gene. Transcripts for all three genes were found in all tissues with the exception of A3A in the brain (Figure 2B). Expression of rA3Z2Z3 and rA1 were tightly related.

### Rabbit A3 localization and function

**Figure 3:**
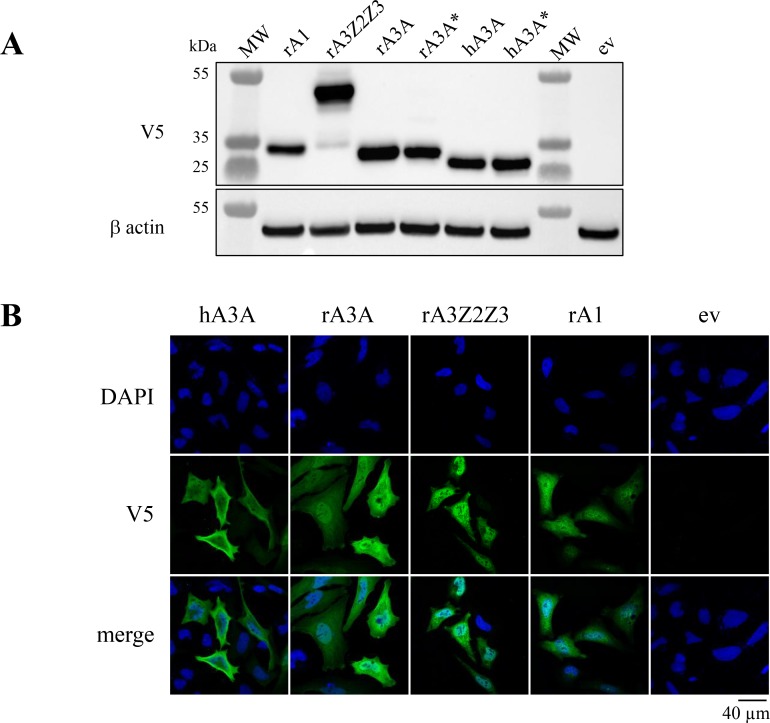
Rabbit A3 proteins show nucleo-cytoplasm localization (**A**) Western blot detection of V5-tagged APOBEC3 proteins in HEK-293T cells. MW, molecular weight; ev, empty vector. β actin probing was used as a loading control. (**B**) Confocal microscopy analysis of V5-tagged A3A proteins in HeLa cells 24 hours post transfection. Nuclei are stained with DAPI.

To characterize these rabbit enzymes, cDNA clones where synthesized for rA3A and rA3Z2Z3 along with rabbit rA1. All three constructs were stable pcDNA3.1/V5-His-TOPO vector unlike some A3 constructs where expression proved to be toxic for bacteria [28]. Human A3A was used as positive control while an inactive catalytic mutant of rA3A was made as a negative control (rA3A^*^). All constructs were well expressed as assessed by Western blot (Figure 3A). Confocal microscopy analysis of transfected HeLa cells indicated a nucleo-cytoplasmic expression pattern for rA3A, rA3Z2Z3 and rA1 (Figure 3B).

**Figure 4:**
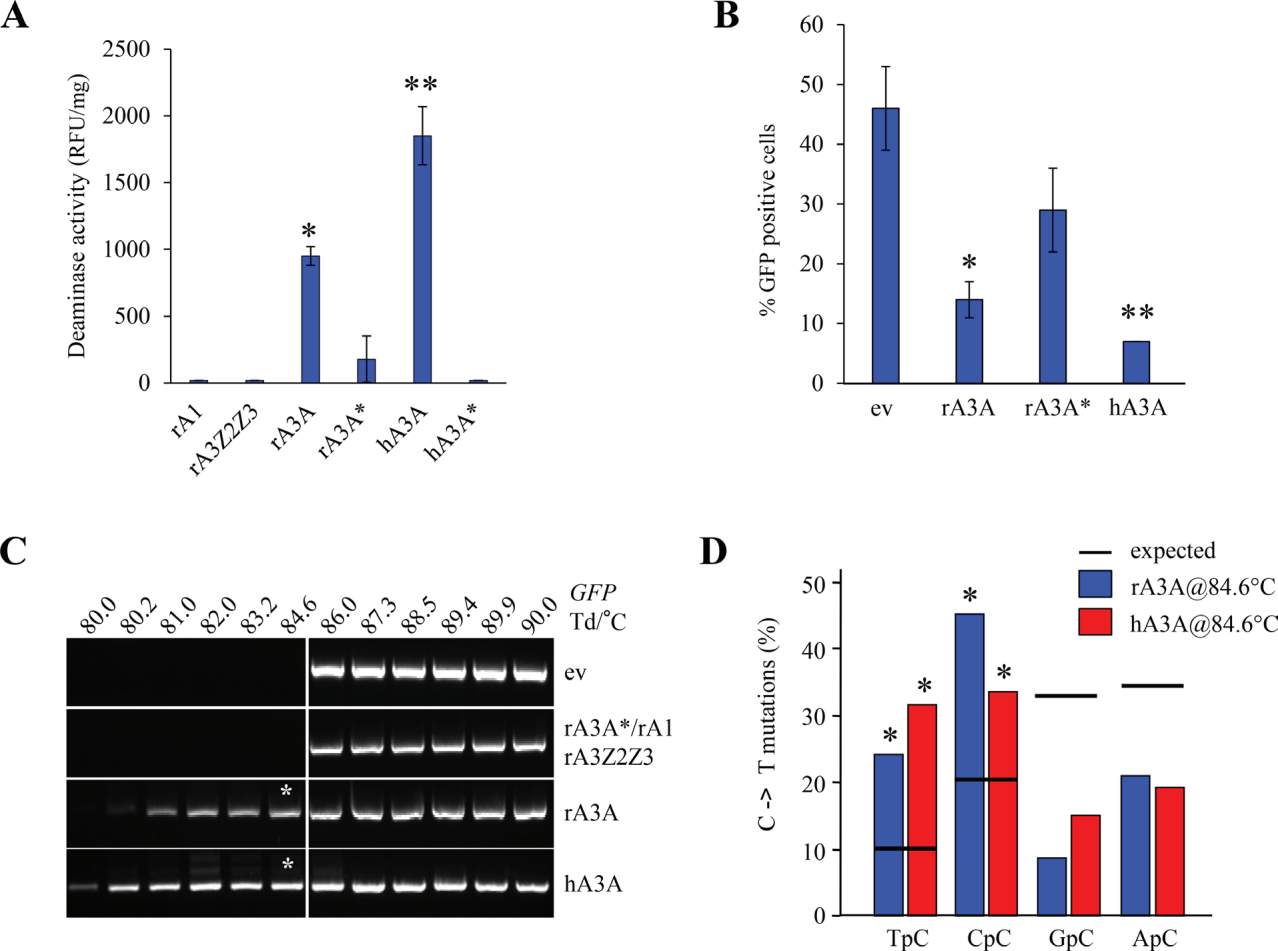
Rabbit A3A deaminates foreign DNA (**A**) Fluorescence resonance energy transfer assay (FRET)-based *in vitro* deamination assay performed on FAM-TAMRA coupled oligonucleotides using transfected HEK-293T lysates. rA3A^*^ and hA3A^*^ catalytic mutants transfected cells used as negative controls. Results are expressed in Relative Fluorescence Unit per mg of protein (RFU/mg). A single asterisk represents a statistically significant difference (*p* < 0.05) and a double asterisk represents a highly statistically significant difference (*p* ≤ 0.005). **(B**) FACS analysis of HeLa cells 72 hours post transfection with an empty vector (ev) or rabbit A3A proteins. Percentage of GFP positive cells are gated on A3-V5-Tag positive cells. Single asterisks represent a statistically significant difference (*p* ≤ 0.05) and double asterisks represent a highly statistically significant difference (*p* ≤ 0.005). (**C**) GFP plasmid 3D-PCR gels from QT6 cells, 48 hours post co-transfection with GFP encoding plasmid and either an empty or an APOBEC3 expressing plasmid. Numbers indicates the Td for each well. Vertical white bar indicates restrictive Td. White asterisks denote PCR products that were cloned and sequenced. (**D**) Analysis of the 5′ nucleotide context of C->T transitions identified in cloned GFP DNA 3D-PCR products. Percentages are represented in blue for rA3A and red for hA3A. Horizontal bars represent expected values. Asterisks show a statistically significant difference (*p* < 0.05).

The activity of rabbit APOBEC enzymes was assessed *in vitro* using a FRET based deamination assay as previously described [28]. No deaminase activity on ssDNA was observed after transfection of HEK-293T cells with rA3Z2Z3 and rA1 (Figure 4A). By contrast, rA3A and hA3A deaminate efficiently the ssDNA well above the background level provided by the inactive catalytic mutants rA3A^*^ and hA3A^*^. To explore DNA editing in quail (QT6) cells which are naturally devoid of any endogenous APOBEC [37], QT6 cells were co-transfected with the A3 constructs along with a GFP reporter plasmid. Reduced GFP fluorescence due to editing of plasmid DNA was first sought by a flow cytometry. As can be seen from Figure 4B, there were more than 2-fold less GFP positive cells in rA3A co-transfected cells compared to the empty vector (ev) (*p* = 0.03) (Figure 4B) and consistent with transfection frequencies between 40–50%. 3D-PCR was used to recover A3 edited DNA which melts at a lower denaturation temperature (Td) [38]. No PCR products were recovered below Td = 86° C when empty vector, rA3A^*^, rA1, or rA3Z2Z3 plasmids were used (Figure 4C). By contrast, products were obtained at temperatures as low as 80° C and 80.2° C with hA3A and rA3A respectively, *i.e.* approximately 6° C below the restrictive Td, this being *prima face* evidence of editing. Cloning and sequencing of products obtained at 84.6° C (represented by an asterisk) confirmed that indeed cytidine deamination occurred with a clear preference for 5′TpC and 5′CpC dinucleotide contexts (Figure 4D).

### Rabbit A3A is functionally orthologous

**Figure 5:**
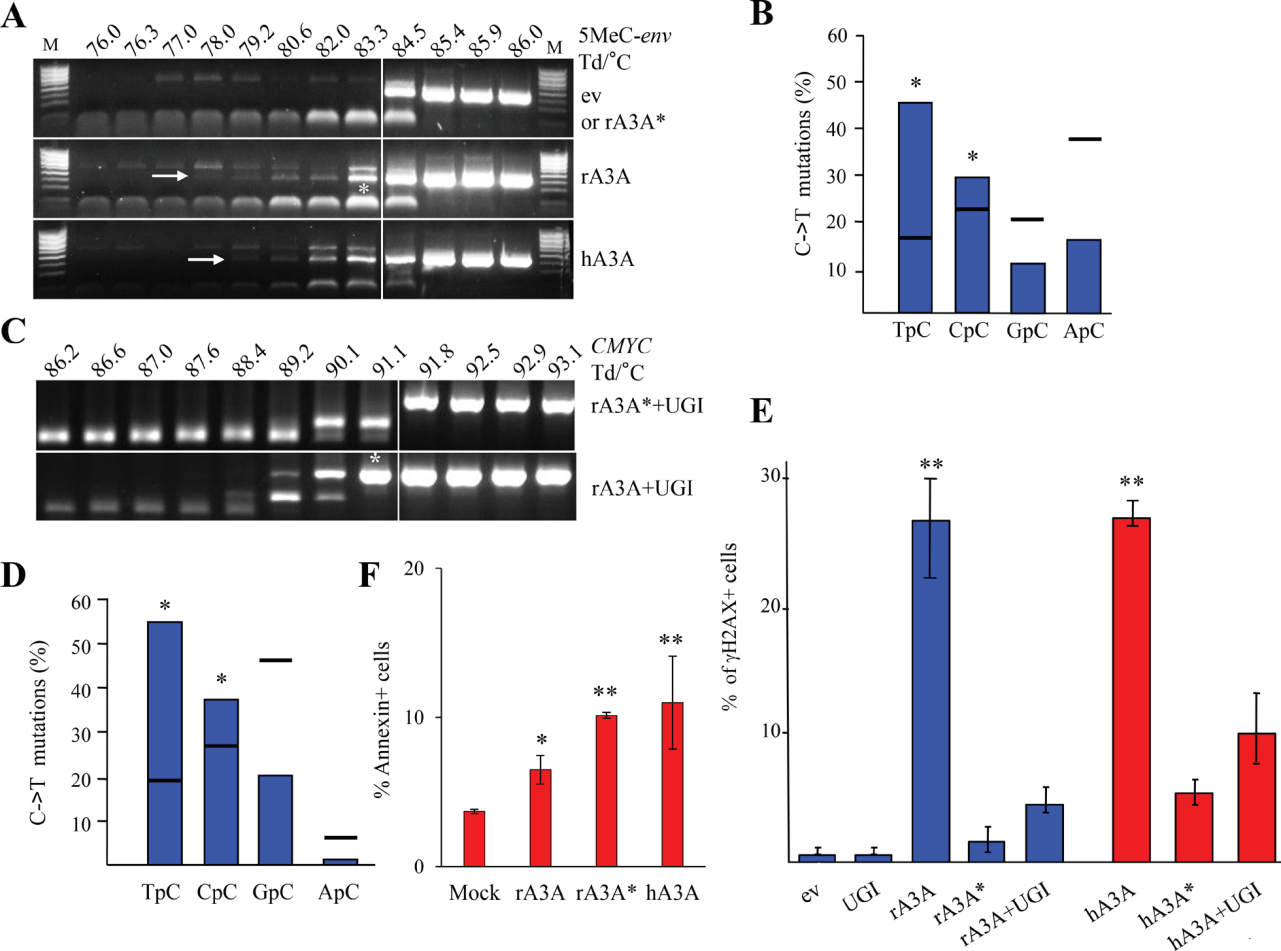
Rabbit A3A edits methylated DNA and generates DBSs driving cells to apoptosis (**A**) QT6 cells were co-transfected with 5-methylated HIV *env* DNA and A3 constructs and analyzed at 48 hours by 3D-PCR. Numbers above gels indicate the Td for each well. Vertical white bar indicates restrictive Td. White asterisk denotes 3D-PCR products cloned and sequenced. (**B**) Analysis of the 5′ nucleotide context of C->T transitions identified in cloned methylated HIV *env* DNA 3D-PCR products. Horizontal bars denote expected values derived from base composition. Asterisks denote a statistically significant difference (*p* < 0.05). (**C**) Quail *CMYC* DNA 3D-PCR gels derived from co-transfected QT6 cells at 48 hours first with UGI and then with rA3A or rA3A^*^ expressing plasmid. Numbers indicate the Td for each well. White asterisk denotes PCR products cloned and sequenced. (**D**) Analysis of the 5′ nucleotide context of C->T transitions identified in A3 edited quail *CMYC* sequences. Percentages correspond to results obtained after rA3A + UGI co-transfection. Horizontal bars represent expected values derived from base composition. Asterisks show a statistically significant difference (*p* < 0.05). (**E**) FACS analysis of HeLa cells 48 hours post transfection with empty vector (ev), UGI or A3A constructs alone or in combination. Percentage of γH2AX positive cells are gated on V5 positive cells except for mock and UGI transfections. Double asterisks mean a highly statistically significant difference (*p* ≤ 0.005). (**F**) FACS analysis of HeLa cells 72 hours post transfection. Annexin positive cells were gated on V5-Tag positive cells, except for the mock condition. Single asterisks represent a statistically significant difference (*p* ≤ 0.05) and double asterisks represent a highly statistically significant difference (*p* ≤ 0.005).

One of the singular traits of mammalian A3A or A3B deaminases is their ability to efficiently deaminate 5MeC and small 5-substituted cytidine oxidation products [22, 25–27]. To demonstrate that rA3A can deaminate 5MeC, QT6 cells were transfected with rA3A expression plasmid and subsequently transfected by 5MeC-substituted HIV *env* DNA fragments [26]. As shown in Figure 5A, 3D-PCR products were recovered as low as 78° C and 79.2° C for the rA3A and hA3A co-transfections respectively, compared to 84.5° C for the empty vector or rA3A^*^ (Figure 5A). The 83.3° C 3D-PCR products (Figure 5A, lower band with an arrow and indicated by an asterisk) were cloned, sequenced and confirmed the presence of edited 5MeC in the expected 5′TpC and 5′CpC dinucleotide context (Figure 5B). In the same rA3A transfected QT6 cells, edited cytochrome c mtDNA in the cytoplasm was also recovered again with the 5′TpC and 5′CpC context (Supplementary Figure 3A and 3B) which is in keeping with the nucleo-cytoplasmic localization of rA3A and published findings for other mammalian A3A enzymes [28].

A3A and A3B are the sole enzymes able to target chromosomal DNA. This can be demonstrated experimentally only if the highly active UNG enzyme is inhibited [20]. As shown in Figure 5C, when rA3A was co-transfected in QT6 cells along with an expression plasmid encoding an UNG inhibitor (UGI), edited *CMYC* DNA was recovered by 3D-PCR at temperatures as low as 89.2° C for the rA3A+UGI compared to 91.8° C for rA3A^*^+UGI (Figure 5C). The 91.1° C 3D-PCR products were cloned, sequenced and confirmed the presence of C->T hypermutation in the 5′TpC and 5′CpC dinucleotide context (Figure 5D).

The ability to generate DSBs is a key feature of all mammalian A3A enzymes tested to date [29, 30]. We used a previously described flow cytometry assay using γH2AX expression as a marker of DSBs [30]. Transfection of rA3A resulted in DSBs on a par with hA3A used as reference while catalytically inactive rA3A^*^ and hA3A^*^ mutants failed to generate DSBs (Figure 5E). Co-transfection with equimolar amounts of UGI strongly reduced the percentage of γH2AX^+^ cells, confirming that DSBs indeed resulted from cytidine deamination and consequent uridine removal by the UNG enzyme during the DNA repair process (r/hA3A versus r/hA3A+UGI, Figure 5E). A3A cytidine deamination of nuDNA can lead to apoptosis (Figure 5F). The proportion of Annexin V positive cells, was ∼2-fold higher in rA3A and rA3A^*^ than in mock transfected cells, and comparable to hA3A (Figure 5F). Once again an A3A catalytically inactive mutant was pro-apoptotic [30] presumably by binding to ssDNA in the nucleus, which could impact the cell cycle leading to cell stress and death.

### Rabbit A3A forms homodimers and is negatively regulated by TRIB3

**Figure 6:**
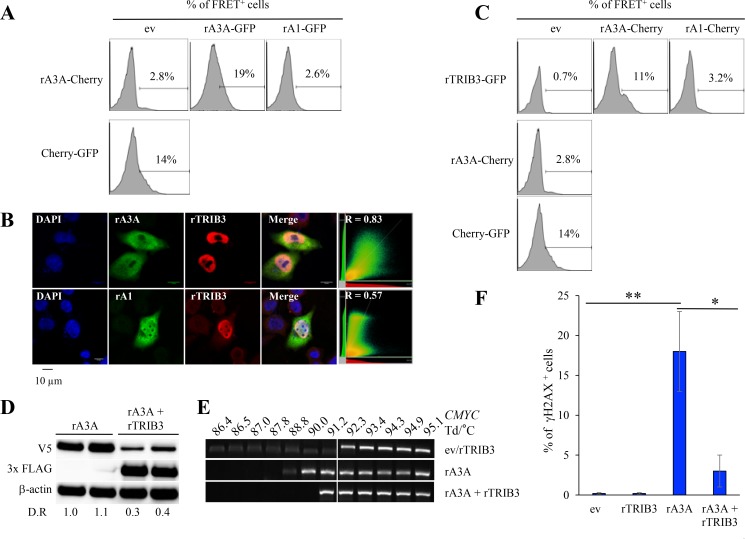
Rabbit A3A forms heterodimers and is negatively regulated by rabbit TRIB3 (**A**) Representative FRET FACS assay of HEK-293T cells 48 hours post transfection with rA3A-Cherry expression plasmid in combination with an empty vector (ev), rA3A-GFP, or rA1-GFP expression plasmid. The percentage of FRET^+^ cells were gated on Cherry^+^ cells for single transfection or Cherry^+^ and GFP^+^ cells for co-transfection. **(B**) Confocal microscopy analysis of V5-tagged APOBEC and 3xFLAG-tagged TRIB3 rabbit proteins in HeLa cells 24 hours post co-transfection. Nuclei are stained using DAPI. (**C**) Representative FRET-FACS assay of HEK293T cells 48 hours after transfection with rabbit APOBEC-Cherry or TRIB3-GFP constructs alone or in combination. A plasmid encoding a Cherry protein fused to a GFP protein was used as a positive control. Percentage of FRET^+^ cells are gated on Cherry^+^ cells for single transfection or Cherry^+^ and GFP^+^ cells for co-transfections. **(D**) Western blot detection of V5-tagged rA3A and 3×FLAG-tagged rTRIB3 proteins in HEK-293T transfected cells by either rA3A alone or rA3A + rTRIB3 (1/4 molar ratio). β actin was used as a loading control. Quantification of fluorescence intensity was performed using ImageJ by normalizing datas to rA3A, D.R, densitometric ratio. **(E**) Human *CMYC* DNA specific 3D-PCR gels from HEK293T-UGI cells 48 hours post transfection with either an empty vector (ev), rA3A alone and rA3A and rTRIB3 together (1/4 ratio). Numbers above the gel indicates the Td for each well. White bar indicates restrictive Td. (**F**) FACS analysis of HeLa cells 48 hours after transfection with an empty vector (ev), rTRIB3, rA3A and rA3A and rTRIB3 together (1/4 ratio). Percentage of γH2AX^+^ cells are gated on V5-Tag positive cells except for mock and rTRIB3 conditions. A single asterisk means the difference is statistically significant (*p* ≤ 0.05) and a double asterisk means the difference is highly statistically significant (*p* ≤ 0.005).

Regulation of hA3A editing is crucial considering its deleterious effect on genome integrity. Many A3 enzymes can homo- and heterodimerize including A3A [39–41]. We investigated rA3A ability to dimerize using a flow cytometry-based Försters resonance energy transfer assay (FRET-FACS). The test was based on an energy transfer from an excited donor fluorophore (a GFP-fusion protein) to a juxtaposed acceptor fluorophore (a Cherry-fusion protein), which results in specific fluorescence emission detectable by flow cytometry. Plasmids containing rA3A or rA1 fused to Cherry or GFP were constructed and transfected alone or in combination in HEK-293T cells. As shown in Figure 6A, when rA3A-Cherry and rA3A-GFP fusion plasmids were co-transfected, the percentage of fluorescent cells in the FRET channel was ∼7-fold (19%) higher over background fluorescence. By contrast, cells transfected with rA3A-Cherry alone gave a background fluorescence (2.8%) whereas transfection with a positive control plasmid encoding a Cherry-GFP fusion construct generated ∼5-fold more fluorescence (14%). That the gain of fluorescence resulted from a *bona fide* specific interaction between rA3A fusion proteins was checked by co-transfecting rA3A-Cherry with the non-homologous rA1-GFP. Only background fluorescence levels were noted due to the inability of the proteins to interact (2.6%).

We then explored whether rA3A interacts with the rTRIB3 pseudokinase protein given that the human counterpart (hTRIB3) is the only human A3A interactor identified for the moment [32]. The *rTRIB3* gene harbors the same exon/intron structure as the human gene with protein sequences displaying 78% identity. Transfection of a synthetic V5 tagged rTRIB3 expression construct in HeLa cells revealed rTRIB3 to be strictly nuclear (Figure 6B), like its human counterpart [32]. Co-transfection experiments showed that rA3A and rTRIB3 co-localized, as assessed by a Pearson’s correlation coefficient test (*R* = 0.83) (Figure 6B). As expected, no co-localization was observed with rA1. To confirm that colocalization corresponded to a *bona fide* interaction between rA3A and rTRIB3 proteins, a rabbit TRIB3-GFP construct was generated to be used in a FRET-FACS analysis as described in Figure 6A. As shown in Figure 6C, the rA3A-Cherry/rTRIB3-GFP co-transfection generated fluorescence ∼15-fold higher (11%) than background (0.7%) or rA1-Cherry/rTRIB3 co-transfection (3.2%).

Transfection of HEK-293T cells with rA3A alone or in combination with rTRIB3 at a 4/1 ratio resulted in ∼3–4 fold decrease in rA3A protein in the presence of rTRIB3 as shown by Western blot quantification (Figure 6D). HEK-293T-UGI cells, which constitutively express the UGI, were cotransfected by rA3A expression plasmids with and without rTRIB3. Transfection of rA3A alone allowed detection of edited *CMYC* DNA. PCR products where obtained down to 88.8° C, *i.e.* 3.5° C below the restrictive Td (92.3° C) (Figure 6E). However, when rA3A + rTRIB3 were co-transfected, 3D-PCR recovered DNA only down to 91.2° C, *i.e.* 2.4° C higher, demonstrating that rTRIB3 expression inhibited rA3A editing of *CMYC* DNA (Figure 6E). Finally, the percentage of γH2AX^+^ in HeLa cells was drastically reduced when cells where co-transfected with rA3A + rTRIB3 compared to rA3A alone (18% versus 3% *p* = 0.003, Figure 6F). Both results indicated that the ability of rA3A to induce mutations and DSBs in genomic DNA was curtailed by rTRIB3.

### Cytidine deamination *in vivo*

**Figure 7:**
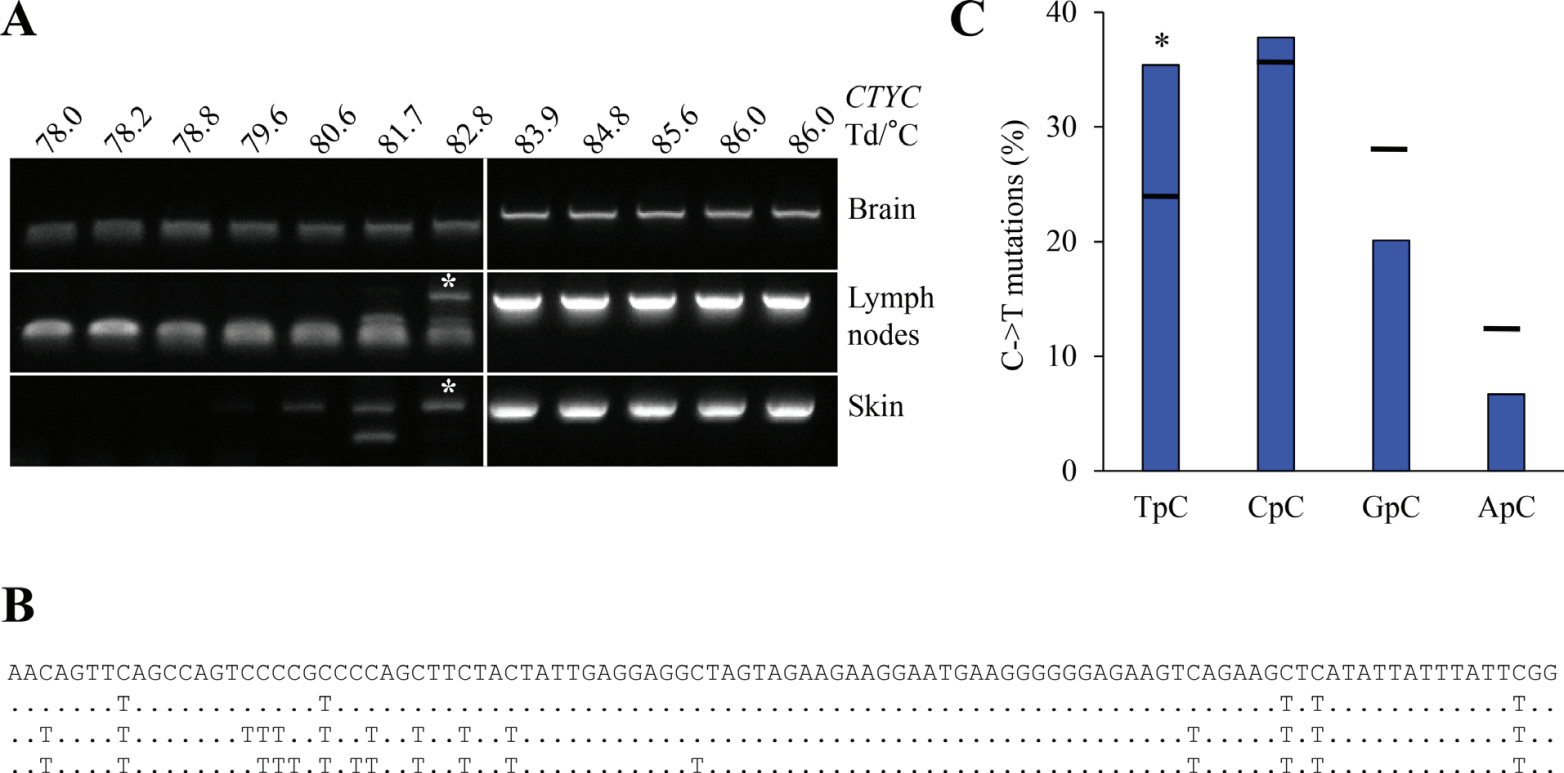
A3 edited mtDNA *in vivo* (**A**) Rabbit cytochrome c mtDNA 3D-PCR gels using total DNA from brain, lymph node and skin tissues. Numbers indicates the Td for each well. White bar indicates restrictive Td. White asterisks indicate cloned PCR products for sequences analysis. If rA3A is orthologous to hA3A then the edited mtDNA is derived from mtDNA leaked to the cytoplasm. (**B**) Representative mutated mtDNA sequences harboring C->T substitutions. Mutations are noted compared to reference sequence. (**C**) Analysis of the 5′ nucleotide context of C->T transitions identified edited cytoplasmic mtDNA. Horizontal bars represent expected values. Asterisks show a statistically significant difference (*p* < 0.05).

The above data showed that only rA3A was a functional as an efficient ssDNA cytidine deaminase in experimental settings (Figure 4 and Figure 5). As *rA3A* was widely expressed *in vivo* (Figure 2B), we sought edited cytoplasmic mtDNA (cymtDNA) in rabbit tissues. Using a nested PCR/3D-PCR protocol [38] focused on rabbit mtDNA *CYTC,* we found PCR products below the limiting Td = 83.9° C from lymph node and skin derived total DNA (Figure 7A). By contrast, nothing was recovered from brain derived DNA where *A3A* transcripts were not detected (Figure 2B). Cloning and sequencing of lymph nodes and skin 3D-PCR products obtained at 82.8° C (see asterisks, Figure 7A) confirmed multiple C->T substitutions (Figure 7B) with a preference for editing in a 5′TpC dinucleotide context, typical of A3A enzymes (Figure 7C). This suggests that only the A3A enzyme is active *in vivo*.

## DISCUSSION

The *APOBEC3* locus is conserved among most placental mammals and usually encodes at least one Z1, Z2 and Z3 zinc-finger containing enzyme [5]. The notable exception is the entire order *Rodentia* that lacks a Z1 containing domain typified by the human A3A enzyme. The pig and cat genomes are also lacking a Z1 domain enzyme whereas the sheep and dog genomes do [28, 33]. The rabbit genome encoded two *APOBEC3* genes in a head-to-head manner, reminiscent of the *Carnivora APOBEC3* locus (Figure 1). The rAZ1 (rA3A) enzyme proved a *bona fide* member as it encodes the signature I128 residue (Supplementary Figure 2A) [28, 42, 43]. To date only the Z1 domain enzymes, notably human A3A and A3B, have been linked to cancers [3, 20, 22, 44, 45]. It has been suggested that one of the human A3H haplotypes might also be an endogenous mutagen [46] although A3H is a Z3 monodomain cytidine deaminase [5]. As the rA3A, A3 enzyme proved to be most closely related to primates, A3A enzyme opens up the perspective of using the rabbit as a small animal model, especially as rA3A was functional (Figures 4, 5 and 6). By contrast, the rabbit A3Z2Z3 protein was stable but functionally inactive (Figure 4A and 4C), using highly sensitive 3D-PCR, a technique that allows recovery of a single hypermutated sequence down to frequencies of 10^–4^ [37, 38]. Catalytically inactive enzymes that are nonetheless expressed *in vivo* have already been described [47, 48]. Human A3DE is an example as the protein is stably expressed, yet non-functional due to a single inactivating amino acid substitution [49]. Alternatively, it could result from an inactive polymorphism. No ssDNA hyperediting activity was detected with rA1 despite reports showing that rat A1 targets HIV DNA albeit much less so than for RNA [50]. This contrasts with murine A1, which can hypermutate both dsRNA and ssDNA [36]. 3D-PCR is excellent for detecting hyperedited ssDNA but is unable to recover DNA with a handful of edited cytidine bases per 100 bp.

Exploration of the cellular localization demonstrated a nucleo-cytoplasmic expression for the three rabbit proteins (rA1, rA3A, rAZ2Z3, Figure 3B). The expression of rA3A is the same for all mammalian A3A studied to date [28, 43]. By contrast, the nucleo-cytoplasmic localization of rA3Z2Z3 makes it different from murine A3 that was strictly cytoplasmic [36, 51, 52]. The rA1 expression pattern was consistent with a previous report [51].

The rabbit A3A enzyme was strictly orthologous to its human counterpart. Its localization was nucleo-cytoplasmic, able to hyperedit cytidine and 5-methylcytidine residues in foreign DNA substrates (Figure 5A), as well as able to access and deaminate nuDNA and induce DSBs (Figure 5C and 5E). Indeed, these are features of all mammalian A3A [25–30]. In addition, rA3A appeared to be similarly regulated. The ability of A3A to form homodimers is conserved in rabbits and the enzyme directly interacts with rTRIB3, the rabbit homolog of human TRIB3 that is the only A3A interactor identified so far [32]. As for the hA3A/hTRIB3 pair, rTRIB3 enhances rA3A degradation, this being followed by a decrease in rA3A editing activity (Figure 6).

The lemur and rabbit genomes are among the very few species that encode vestiges of endogenous lentiviruses [53, 54]. All but one of the exogenous lentiviruses encode a *vif* gene that neutralizes several, but not necessarily all, APOBEC3 enzymes. While the lemur endogenous retroviruses encode a *vif* gene, the rabbit elements referred to as RELIKs do not encode a *vif* gene [53, 54]. The present work suggests that the infectious counterpart was not vulnerable to restriction by the only functional rA3A enzyme, although other features could allow a virus to replicate despite the presence of APOBEC3 enzymes; for example human hepatitis B virus is vulnerable to editing by human APOBEC3 enzymes yet does not encode an APOBEC3 or interferon antagonist [14, 16, 55–57] given that several human APOBEC3 genes are interferon stimulated genes [32, 58–61].

The results presented here showed that the rabbit genome encodes an A3A enzyme that is strictly homologous to the human A3A in terms of substrate specificity and negative interaction with TRIB3. Moreover, the conservation of the relationship between A3A and TRIB3 over 100 million years, the time between the divergence of *Glires* and *Primates*, illustrates the importance of regulating genomic DNA editing. As all suborders in *Rodentia* do not encode an *A3A* gene, the rabbit thus appears as the only small available animal model for studying the role of A3A during human oncogenesis. However, *rA3A* is widely expressed (Figure 2B). By contrast the *hA3A* is preferentially expressed in hematopoietic cells and is massively up regulated by type I interferons [32, 59, 61]. This suggests that although rA3A is strictly orthologous to its human counterpart, and indeed to other mammalian A3A enzymes, it is differently regulated.

## MATERIALS AND METHODS

### Plasmids

Rabbit A3A, A3Z2Z3 and TRIB3 (NC_013672) cDNAs were synthetized (GeneCust) and subsequently cloned into pcDNA3.1/V5-His-TOPO vector (Invitrogen). All constructs were C-tagged by the V5 epitope excepting for the rabbit TRIB3 construct which was tagged with the 3XFLAG epitope in N-terminal position. The rabbit A3A catalytic mutant rA3A C98S (rA3A^*^) was generated using the GENEART^®^ site-directed mutagenesis system. The hA3A, hA3A catalytic mutant, hTRIB3-3XFLAG and UGI plasmid have been previously described [20, 32]. The Cherry-GFP fusion protein encoding plasmid was from Addgene (#32643)

### DNA extraction, 3D-PCR amplification and cloning

Total DNA from transfected cells was extracted using the MasterPure^™^ complete DNA and RNA purification kit (Epicentre) and suspended in 30 µL of sterile water. All amplifications were performed using first-round standard PCR with 5 µL of DNA extract followed by nested 3D-PCR with 5 µL of 1/50 dilution of the first PCR round. PCR was performed in 50 µL with 1 U Taq DNA polymerase (Eurobio) per reaction. 3D-PCR primer sequences are indicated in Supplementary Table 1. PCR conditions for the first round of amplification were 5 min. of denaturation at 95° C then 40 cycles of amplification (30 sec. 95° C, 30 sec. 58° C, 30 sec. 72° C), followed by 7 min. at 72° C. PCR conditions for the second round of amplification were 5 min of denaturation with dT° gradient of 80–90° C then 40 cycles of amplification (30 sec. 95° C, 30 sec. 80–90° C, 30 sec. 72° C), followed by 7 min. at 72° C. For methylated DNA 3D-PCR, primer sequences and PCR conditions were previously documented [26]. For human CMYC DNA 3D-PCR, primer sequences and PCR conditions were previously described [20]. After purification, PCR products were cloned into TOPO 2.1 vector (Invitrogen) and sequencing was outsourced to Eurofin.

### Real time PCR quantification

Rabbit tissues were incubated in RNA later (RNA stabilization reagent, Qiagen) and mechanically disrupted before extraction of total RNA using RNeasy^®^ lipid tissue mini kit (Qiagen) according to the manufacturer’s protocol. Corresponding cDNAs were synthetized using QuantiTect reverse transcription kit (Qiagen). Quantification was performed by TaqMan using Takyon Rox probe mastermix dTTP blue (Eurogentec). Sequences of specific primers and probes used are detailed in Supplementary Table 1. Cycling conditions were as follows: first step of denaturation at 95° C during 3 min., followed by 40 cycles of amplification (95° C 15 sec, 58° C 15 sec and 68° C 15 sec). Fluorescence was measured during the 68° C step incubation using a Realplex^2^ Mastercycler (Eppendorf). The specificity of the PCR products was verified by sequencing. Messenger RNA expression levels were normalized based on the EF1α reporter gene.

### Cell culture

Japanese quail embryonic fibroblast QT6 cells (ATCC CRL 1708) were maintained in Ham’s medium supplemented with 1% chicken serum, 10% fetal bovine serum, 5% tryptose phosphate, 2 mM L-glutamine, 100 U/ml penicillin and 100 mg/ml streptomycin. HeLa, HEK-293T and HEK-293T-UGI cells (HEK-293T cells stably expressing *Bacillus subtilis* phage uracil-DNA glycosylase inhibitor or UGI) were maintained in DMEM glutamax medium (Invitrogen) supplemented with 10% FCS, 100 U/ml penicillin and 100 mg/ml streptomycin.

### Cell transfection

Approximately 800,000 cells were seeded into 6-well plates and transfected with 2 µg of plasmid using the Jetprime transfection kit (Polypus Transfection™) according to manufacturer’s instructions. For cotransfections, a plasmid ratio of 1:1 was used except for rTRIB/rA3A cotransfection experiments where a ratio of 4:1 was used.

### Deamination assay

At 48 hours post-transfection, A3-transfected HEK-293T cells were extensively washed with PBS and mechanically harvested. Total proteins were extracted using specific lysis buffer (25 mM HEPES [pH 7.4], 10% glycerol, 150 mM NaCl, 0.5% Triton X-100, 1 mM EDTA, 1 mM MgCl_2_, 1mM ZnCl_2_) supplemented with protease inhibitors. Deaminase activity was assessed by incubating whole cell lysates with 1 pmole DNA oligonucleotide 5′FAM-AAATTCTAATAGATAATGTGA-TAMRA in the presence of 0.4 U UDG (New England Biolabs) in a 20 mM Tris-HCl pH7, 1 mM dithiothreitol, and 1 mM EDTA reaction buffer. After 2 hours of incubation at 37° C, abasic sites were cleaved by heating for 2 min at 95° C, and endpoint fluorescence were measured using a Realplex 2 mastercycler (Bio Rad) with FAM setting. Results are normalized to the quantity of protein using Pierce BCA protein assay kit (Thermo Scientific).

### Immunofluorescence

Approximately 50,000 HeLa cells were seeded in Nunc™ Lab-Tek™ II Chamber Slide™ System Thermo Scientific™ and transfected 24 hours later with 1 µg of plasmid DNA according to the Fugene^®^ protocol. Two days after transfection, coverslip grown transfected HeLa cells were washed three times with PBS and fixed with 4% PFA for 15 min. Cells were then washed five times and permeabilized with a 50% methanol/acetone mix for 10 min. After five PBS washings, permeabilized cells were incubated for 1 hour at room temperature, first with 1% bovine serum albumin (BSA) PBS 1/200 mouse monoclonal anti-V5 antibody (Invitrogen) and then with 1% BSA PBS 1/750 anti mouse Alexa Fluor 488 conjugated antibody. After several PBS washings, coverslips were mounted with Mounting medium for immunofluorescence (Interchim). Imaging was performed using a Leica SP5 confocal microscope. Colocalization analyses were performed with Huygens Essential for Mac version 4.3.1p3 (Scientific Volume Imaging B.V.). Images were first deconvoluted then analyzed using the Costes method.

### FACS analysis

For restriction of GFP expression plasmid experiments, transfected cells were trypsinized, washed with PBS, fixed in 2% ice-cold paraformaldehyde (Electron Microscopy Sciences) for 10–20 min. After one PBS washing, cells were permeabilized in 90% ice-cold methanol (Sigma) for 30 min. Following the second PBS washing, cells were incubated for one hour in ice with 1:100 PBS-0.5% BSA diluted mouse anti V5-Tag Alexa Fluor^®^ 647 antibody (AbD Serotec). For apoptosis experiments, cells were first stained using an Annexin V Staining with Fixable Viability Dyes kit (eBioscience) using fixable Viability Dye eFluor^®^780 according to manufacturer’s instructions, then washed, permeabilized and incubated for one hour in ice with 1:100 PBS-0.5% BSA diluted mouse anti V5-Tag Alexa Fluor^®^ 488 antibody (AbD Serotec). For DSBs experiments, fixed and permeabilized cells were incubated 1 hour in ice with 1:100 PBS-0.5% BSA diluted mouse anti V5-Tag Alexa Fluor^®^ 488 antibody (AbD Serotec) and 1:100 diluted mouse anti human γH2AX and 1:100 Alexa Fluor^®^ 647 antibody. For the FRET FACS assay, cells were simply trypsinized and washed with PBS. The data was acquired on a MACSQuant^®^ analyzer harboring violet, blue, and either a red laser (measure of dsDNA breaks and apoptosis) or a yellow laser for the FRET FACS assay. The data were analyzed using the FlowJo^®^ software (Tree Star Inc., version 10.1r5 for Mac).

### Western blotting

Cells were recovered 24 hours after transfection. Protein extraction and Western blot analysis were carried out according to standard procedures. After blocking, membranes were probed with either a 1:5000 dilution of anti V5-tag horseradish peroxidase-coupled antibody, a 1:5000 dilution of anti 3x-FLAG peroxidase-coupled antibody (Sigma), or a 1:50000 dilution of anti β-actin. The membrane was subjected to detection by SuperSignal™ West Pico chemiluminescent substrate (ThermoFisher Scientific).

## SUPPLEMENTARY MATERIALS FIGURES AND TABLE


