# Supplementary material for: The rabbit as an orthologous small animal model for APOBEC3A oncogenesis
Source: Oncotarget. 2018 Jun 12;9(45):27809–22. doi: 10.18632/oncotarget.25593 (PMC6021247; doi:10.18632/oncotarget.25593)
Supplement: Supplementary file 1 [file oncotarget-09-27809-s001.pdf]

The rabbit as an orthologous small animal model for APOBEC3A oncogenesis

SUPPLEMENTARY MATERIALS

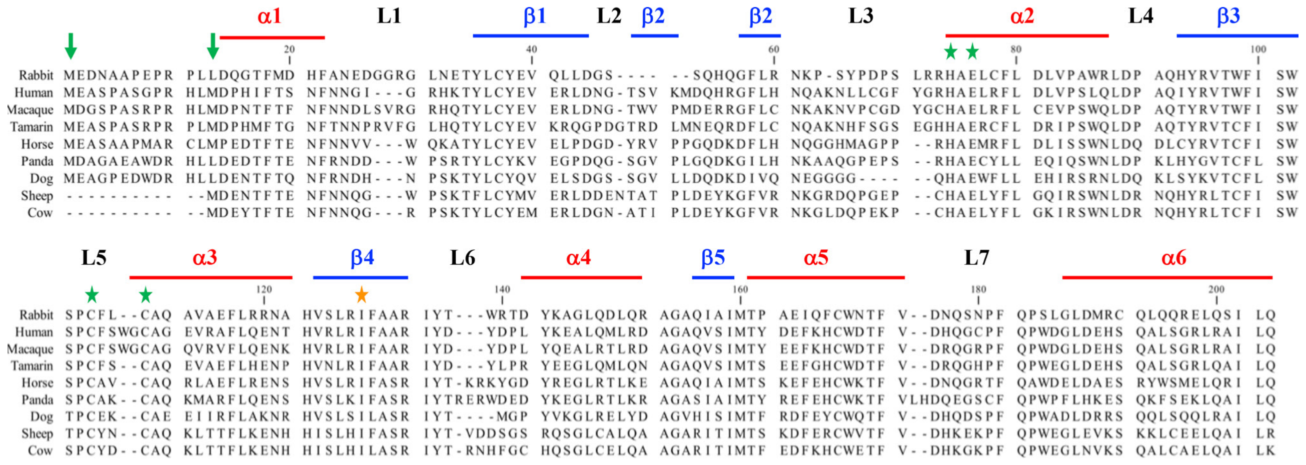

Supplementary Figure 1: Comparison of A3A proteins. Alignment of rabbit A3A protein sequence with those of eight mammalian A3A sequences spanning exons 1–4. Exon 5 sequences are so varied they were not included in the analysis which was constructed using MCC Main Workbench 7.0.2 software. Green arrows indicate the two alternative start codons generating the two distinct hA3A isoforms. Green asterisks indicate key residues involved in zinc coordination responsible for enzymatic activity. Orange asterisks indicate the Z1 specific amino acid I128. Structural motifs structures (α helix, β sheet and loop) are indicated.

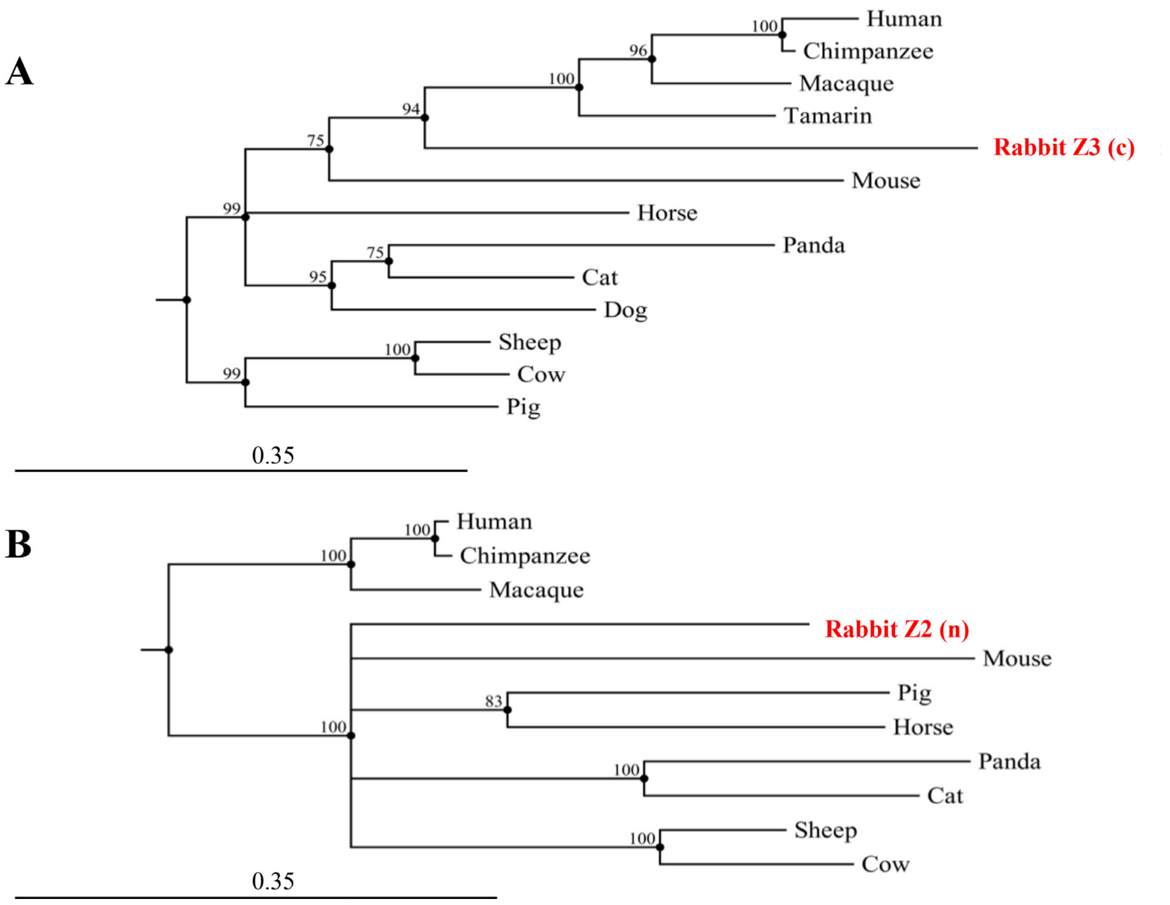

Supplementary Figure 2: The Z2Z3 enzymes is only distantly related to mice A3. (**A**) Phylogeny of mammals Z3 catalytic domain protein sequences constructed using the Neighbor-joining method with the MCC Main Workbench 7.0.2 software. Numbers correspond to bootstrap values inferred from 1,000 replicates. Bootstrap values under the threshold of 70% are not shown. (**B**) Phylogeny of mammals Z2 protein sequences constructed using the Neighbor-joining method with the MCC Main Workbench 7.0.2 software. Numbers correspond to bootstrap values inferred from 1,000 replicates.

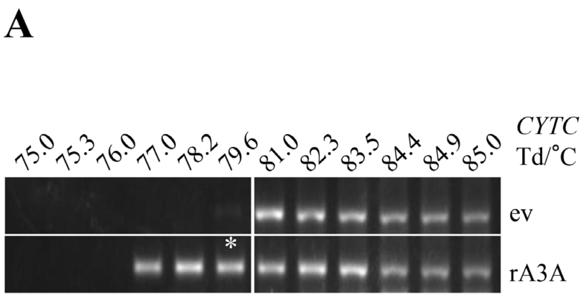

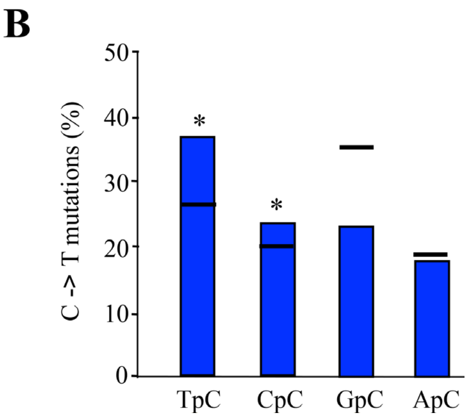

Supplementary Figure 3: Rabbit A3A edits cytochrome c mitochondrial DNA after transfection *in vitro*. (**A**) Quail *CYTC* mtDNA 3D-PCR gels from QT6 cells 48 hours after transfection with an empty vector (ev) or rA3A expressing plasmid. Numbers indicates the Td for each well. The white bar indicates the restrictive Td. A white asterisk denotes amplicons that were cloned and sequenced. (**B**) Analysis of the 5′ nucleotide context of C->T transitions identified in cloned quail *CYTC* mtDNA sequences. Percentages correspond to results obtained after rA3A transfection. Horizontal bars represent expected values. Asterisks show a statistically significant difference ($p < 0.05$).

Supplementary Table 1: Primers and probes used for amplification of plasmid DNA by 3D-PCR and real time PCR quantification of rabbit mRNA

OUT-F	5′-TAGTTGCCTGACTCCCCGTCGTGTAGATAA-3′
OUT-R	5′-TGACAACGATCGGAGGACCGAAGGAGCTAA-3′
IN-F	5′-ACGCTCACCGGCTCCAGATTTATCAGCAA-3′
IN-R	5′-TTGATGGTTGGGAACCGGAGCTGAATGAA-3′
EF1α-F	5′-TGTTGAGAGCTTCTCTGACTATCC-3′
EF1α-R	5′-CTCCAGCAGCCTTCTTGTCC-3′
EF1α-P	5′-JOE-CCTCTGGGTCGTTTCGCTGTCCGT-BHQ1-3′
rA1-F	5′-CCGAGAAAGGTCCTTCAAAC-3′
rA1-R	5′-GGCCTCTTTACGCAGTTCTT-3′
rA1-P	5′-FAM-TCCCAGGGTTCAATTCTTCTCCTCA-TAMRA-3′
rA3Z2Z3-F	5′-GGTGAAGAATCTGCAGGACA-3′
rA3Z2Z3-R	5′-CTGGTTGTTGAACTGGAAGC-3′
rA3Z2Z3-P	5′-FAM-CCTCGAGCAGACCCTTGGCG-TAMRA-3′
rA3A-F	5′-CGCAACAAGCCTAGTTATCCA-3′
rA3A-R	5′-AGGTCCAGGAAACACAGCTC-3′
rA3A-P	molecular probe #70 (Roche)
